# *Vorvida*: study protocol of a randomized controlled trial testing the effectiveness of Internet-based self-help program for the reduction of alcohol consumption for adults

**DOI:** 10.1186/s12888-016-0725-9

**Published:** 2016-01-29

**Authors:** Jördis M. Zill, Björn Meyer, Janine Topp, Anne Daubmann, Martin Härter, Jörg Dirmaier

**Affiliations:** Department of Medical Psychology, University Medical Center Hamburg-Eppendorf, Martinistr. 52, 20246 Hamburg, Germany; GAIA AG, Gertigstraße 12-14, 22303 Hamburg, Germany; Department of Medical Biometry and Epidemiology, University Medical Center Hamburg-Eppendorf, Martinistr. 52, 20246 Hamburg, Germany

**Keywords:** Alcohol, Harmful drinking, Alcohol dependence, *Vorvida*, Online-treatment, Internet-based intervention, Internet self-help, eHealth, Computer-tailoring, Randomized controlled trial (RCT)

## Abstract

**Background:**

Problem drinking is an important global health concern, causing premature mortality and morbidity. Only few problem drinkers seek professional care, unfortunately, because of multiple barriers such as insufficient change motivation, fear of stigmatization or limited access to care. The aim of this study will be to examine the effectiveness of a novel Internet intervention termed *Vorvida*, which was developed based on established cognitive-behavioral therapy techniques with the aim of reducing problematic alcohol consumption.

**Methods/Design:**

A two-arm randomized control trial (RCT) will be conducted to determine whether using Vorvida results in greater reductions in self-reported problem drinking, compared with a care-as-usual/waitlist (CAU/WL) control group. There will be a baseline assessment (t0) and follow-up assessments after three (t1) and six months (t2). Inclusion criteria will be: minimum age of 18, an average consumption of alcohol >24/12 g (men/women) per day and an AUDIT-C score ≥ 3, as well as informed consent. Participants will be randomly assigned to the intervention or control condition at a ratio of 1:1. Recruitment, informed consent, randomization and assessment will be Internet-based. Primary outcome will be change in self-reported alcohol consumption between t0 and t1. Secondary outcomes will be self-reported drinking behavior, expectancies of effects of alcohol use, abstinence and relapse tendencies, self-efficacy and motivation to change.

**Discussion:**

This study is expected to establish the extent to which a novel Internet intervention could contribute to reducing problem drinking among adults with mild to severe alcohol use disorders who may or may not seek or access a traditional treatments. Potentially, this program could be an effective and efficient tool to help reduce problem drinking on a population level because a great number of users can be reached simultaneously without adding burden to treating clinicians.

**Trial registration:**

German Clinical Trial Registration (DRKS): DRKS00006104. Registered 14 April 2014.

## Background

Over recent decades alcohol misuse has become a major public health problem. Problematic alcohol consumption is continuing to increase and the harms associated with it are severe [1]. The World Health Organization (WHO) reported that individuals above 15 years of age consume an average of 6.2 l pure alcohol each year. Major worldwide variation has been reported, with people living in Russia and Eastern Europe consuming the most alcohol [1, 2]. In Germany, consumption is significantly above the global average, with 9.6 l pure alcohol per year and inhabitant [3, 4]. About 3.4 Million Germans meet criteria for harmful drinking or alcohol dependence while at least 10 million people in Germany are considered to be above the threshold of low-risk consumption and, therefore, engage in hazardous drinking [3–5]. Excessive alcohol consumption causes harm on an individual and societal level. It has been identified as being an important cause for premature mortality and morbidity [1]. Furthermore, excessive alcohol consumption can be regarded as an essential risk factor contributing to the global burden of disease and injury. About 4 % of all deaths and 5 % of disability adjusted life years worldwide are associated with harmful use of alcohol [1, 6]. With approximately 26.7 billion euro per year, the economic impact of alcohol-related diseases is substantial [3].

Although a variety of effective interventions are available and have been shown to be effective, only few problem-drinkers seek professional help [7, 8]. Indeed, alcohol abuse and dependence may have the widest treatment gap, compared with other mental disorders, given that 78 % of affected individuals do not receive treatment [9]. Moreover, stigmatization of people with alcohol dependency may be more severe compared to some substance-unrelated mental disorders because those affected are often regarded as more responsible for their condition; they evoke more social rejection and negative feelings, and their risk for structural discrimination is particularly high [10]. Moreover, motivation to change among many of those with harmful alcohol use is notoriously difficult and can function as a treatment barrier.

In order to overcome barriers associated with traditional formats of treatment and extend existing approaches, internet-based interventions have gained popularity over the past decade [11]. Internet-based interventions have the potential of reaching large parts of the population and allow a high degree of flexibility because they can be used at nearly any time or place, given the widespread availability of mobile Internet devices, such as smartphones. Furthermore, barriers such as privacy concerns and fear of being stigmatized or labeled as an alcoholic could be overcome by the anonymity of Internet-based interventions [12–14]. Current findings clearly indicate there is a high demand for Internet interventions in the general public [15]. A current meta-analysis of Riper and colleagues [16] found 16 RCTs on the effectiveness of Internet interventions designed for adult alcohol misuse. The results showed a small but significant overall effect (g = 0.20, 95 % CI: 0.13–0.27, *p* = .001) for the Internet interventions. Previous meta-analysis found slightly higher effects for Internet interventions that targeted other conditions, such as depression [11, 17–19].

Compared to the considerable number of RCTs on disorders such as depression and anxiety, only few RCTs have addressed alcohol disorders [16, 18–21], and most of them included mainly student and young adult samples rather than the general adult population [14, 22]. Moreover, most of the RCTs included in current meta-analyses examined single-session Internet interventions [16, 22], although some authors discuss that more extended interventions could be more effective [11, 23].

In addition, many interventions described in these meta-analysis [11, 16, 22, 23] can be accessed via Internet but are severely limited in the extent to which they use the interactivity and tailoring opportunities afforded by current software technology. Essentially, some Internet interventions consist of little more than conventional self-help text, offered in the same format to all users, without few or no interactive or “responsive” program features or custom-tailoring of content [24]. Programs that use many interactive elements, tailoring of content, and responsive web-design (i.e., self-adjusting layout of a program to the characteristics of the device used, such as desktop, tablet, smartphone), should allow for more flexible use and more user engagement, which in turn might enhance the effectiveness of the intervention. Indeed, meta-analyses have shown that interactive or tailored interventions tend to outperform non-tailored interventions in trials seeking to improve health-related knowledge or change health-related attitudes and behavior [24]. “Computer-tailoring” is a technique of adaptive communication which is described as a “combination of strategies and information intended to reach one specific person based on characteristics that are unique to that person, related to the outcome of interest, and derived from an individual assessment” [25]. Some Internet interventions use this tailoring principle by continuously requesting individual user choices and then adjusting subsequent content, in a manner that simulates an individual “dialogue” between user and the program [26, 27].

The purpose of this study is to test the effectiveness of *Vorvida*, a responsive and tailored Internet intervention against a care as usual (CAU)/waitlist (WL) condition. First, we hypothesize participants in the intervention condition, compared to the control group, will report significantly greater reductions in alcohol consumption at the three month (t1) and six month time-points (t2) compared to CAU, with t1 considered to be the time-point of primary interest. Second, we hypothesize that participants receiving the intervention differ from those in the CAU condition in measures of expectancies of effects of alcohol use, abstinence and relapse, self-efficacy and motivation to change.

## Methods

### Study design

This study is conducted as a parallel-groups randomized controlled trial. Participants will be randomized into two groups: 1) Immediate access to the Internet intervention (*Vorvida* group) and 2) CAU/WL Participants in the first group will receive individual vouchers with which they can immediately begin using the program. The participants in the second group will receive their access vouchers after a delay of six months (that is, after completing the t2 online questionnaires).

### Recruitment

The sample will be recruited through multiple access ways, including health insurance companies (e.g. advertisement in newsletters or websites), care providers (e.g. advertisements at family doctors' offices, clinics, information centers), non-care providers (Internet forums, newspaper adds, panels, print media). Potential participants will be informed that they can visit a website (www.vorvida-studie.de) that contains detailed information about the study. Additionally, participants will be recruited through the German e-mental health portal www.psychenet.de.

### Study procedure

We developed a study website (www.vorvida-studie.de) to inform potential participants about the aim of the study, the procedure, data protection and possible benefits and risks of the study. Participants will be informed that they can withdraw from the study at any time without having to disclose reasons. After reading all information, electronic informed consent will be sought before the screening for eligibility. Persons willing to participate will be asked to fill out an online screening questionnaire to test if they meet inclusion criteria. Within one week, participants will receive an e-mail to inform them whether inclusion criteria are met and, if so, what next steps are required. All included participants will receive a link to the online baseline questionnaire (t0) and are asked to respond to it within one week. An e-mail reminder will be sent out after two and after four weeks to those who did not reply. After responding to the baseline questionnaire, participants will be randomized to one of the two study arms. Each participant will receive an e-mail with the randomization result. The e-mail of the participants randomized to the *Vorvida* group will contain the access key to the *Vorvida* program, a 12 digit number registration number that activates the program for 180 days after initial registration. The CAU/WL group will be informed about the waiting time of six months until they receive their access key to the program.

Three (t1) and six months (t2) after completing the baseline questionnaire (t0), participants in both groups will receive an online-link for the follow-up questionnaires. They will be asked again to respond within one week and will receive two reminder e-mails, after two and after four weeks, if they do not do so. Furthermore, participants in the *Vorvida* group will receive an e-mail reminder to encourage program use after three weeks, six weeks, nine weeks and sixteen weeks after receiving the access key.

A bias of attrition, meaning the loss of participants to the follow-up assessment is a well-known problem in RCTs, particularly of Internet interventions, and can affect the strength of the findings [28]. Because non-monetary incentives have been shown to reduce attrition in online trials [29, 30] each participant who completes the baseline questionnaire (t0) and the two follow-up questionnaires (t1 and t2) will receive a 10€ Amazon gift voucher.

**Fig. 1:**
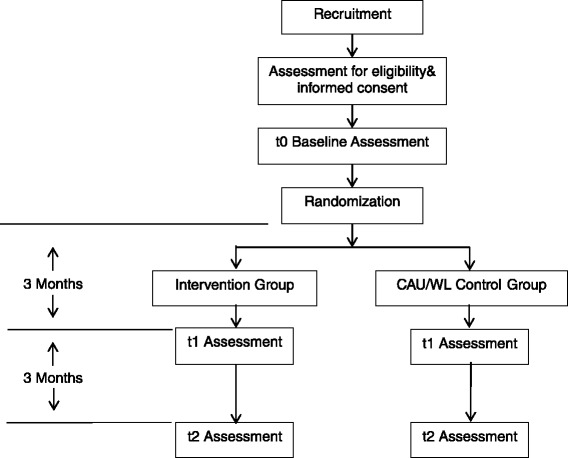
Trail flow of the study.

The trial flow of this study is shown in Fig. 1. The results will be reported in accordance with the CONSORT E-health statement.

### Inclusion and exclusion criteria

Preconditions to participate in the study are: e-mail access, availability of a computer, tablet or a smartphone, access to the Internet, Internet literacy, sufficient knowledge of the German language and no impairments in hearing, speech and vision.

Inclusion criteria are: a minimum age of 18, heavy drinking episodes or harmful and hazardous alcohol consumption, according to guidelines published by BZgA [5] (www.bzga.de) (an average consumption of >24/12 g (men/women) pure alcohol per day and/or an AUDIT-C score ≥ 3) [31], and informed consent. Exclusion criteria will be of suicidal ideation and/or tendency and no informed consent. See Table 1.

**Table 1:** Inclusion and exclusion criteria.

Inclusion criteria	Exclusion criteria
Minimum age of 18	Presence of suicidal ideation and/or tendency
An average consumption of >24/12g (men/women) pure alcohol per day	No informed consent received
AUDIT-C score ≥ 3	
Informed consent received	

### Sample size

The sample size is estimated using Gpower v.3.0.5 software [32]. The calculation is based on the primary outcome measure (alcohol consumption).

Other studies on Internet self-help programs on alcohol misuse showed mainly small effect sizes between d > .2 and d < 0.4 [11, 18].

To detect effect sizes of d = 0.28, an effect derived from a meta-analysis of self-guided Internet interventions [18], with a power of 0.80 and a significance level of 0.05 to compare the *Vorvida* group with the CAU/WL group, a target sample size of N = 404 (n = 202 per group) is required. Since it is expected that 20 % of variance can be explained through baseline-covariance, adjusted effect sizes were used in this power calculation.

### Randomization

After the completion of the baseline questionnaire (t0) participants will be randomized with the ratio 1:1 to the two study arms (intervention or control group). The randomization will be conducted with a computer generated list of numbers. This list is generated by an independent researcher; the other researchers are blinded to the list, ensuring concealed allocation to conditions.

### Intervention

#### Access to the intervention

The participants in the intervention group will receive an access voucher immediately after completing t0 questionnaires, which will enable them to use the *Vorvida* program for approximately six months (180 days) after initial registration.

#### Intervention description

The *Vorvida* program was designed for people aged above 18 who consider their own alcohol consumption patterns to be problematic and are therefore seeking help on the Internet. It is intended for those with harmful and hazardous alcohol consumption patterns as well as alcohol dependence. After initial registration and accepting the program’s terms and conditions, users are asked to enter their e-mail address and set a password, which can then be used to access the personalized program on any suitable device, including smartphones and tablet or desktop computers.

*Vorvida* was produced with the broca® software, which was developed by the GAIA AG and is the basis for several other programs (e.g. deprexis) [26]. Broca uses a computer-tailoring approach in which the program interacts as an “expert” with the user, who continuously responds by choosing from a menu of predetermined options, such that a “simulated dialogue” emerges with every user. Users are addressed by a name they can set for themselves, and an attempt is made in the “dialogues” to provide personally relevant information, with the aim of increasing learning effects [33].

The users can work individually on different contents by reading short text sequences for approximately 15 to 45 min, depending on reading speed and on individual response selections and differing paths through the program content. The program’s content is based on a range of established, evidence-based cognitive-behavioral therapy techniques, which are referenced within the program, as described below. Therefore, the program is operating with different techniques to change behavior techniques, including procedures gleaned from motivational interviewing (e.g., decisional balance), goal setting, self-monitoring of symptoms with questionnaires, cognitive and behavioral strategies for handling alcohol cues, craving and risk situations. Further, cognitive restructuring, mindfulness-based methods, mental imagery and homework exercises are introduced.

The *Vorvida* program is broadly organized in four modules. The first module focuses on individual drinking patterns, e.g. clarification of intrinsic versus extrinsic change motivation [34], exploration of the perceived advantages and disadvantages of drinking [35] and education about the harmful consequences of alcohol abuse. Moreover, realistic goal setting (e.g. reduction of consumption or abstinence) is discussed in this first module. The second module focuses on coping with alcohol craving, e.g. identification of trigger cues and mind-fullness based methods for handling craving (e.g., “urge surfing”), as well cognitive reframing techniques [36]. The third module focuses on coping with risk situations, e.g. distraction techniques [37], imaginative cue exposure [38] and problem-solving techniques [39]. The fourth module focuses on dealing with slips and relapses and summarizes the content of previous modules. It discusses the achieved goals and long-term goals and provides an “emergency case” with cognitive and behavioral methods that might be used in a relapse situation [36].

Moreover, the program includes two short questionnaires, 1) a “mood-check”, which is offered daily to allow the user to monitor and reflect on mood fluctuations over time, 2) a weekly alcohol consumption check that is based on two items: a) “On how many days did you drink alcohol?” (response options: 0–7 days) and b) “How much alcohol did you drink if you drank alcohol on one day?” (response options 1 to 12 standard drinks). Users receive individual feedback on these two items, taking into account both frequency and amount of alcohol consumption, to help them monitor their alcohol use pattern.

Users can engage with the program at their own speed without the need of following a specific schedule. However, based on experiences with previous programs, it is recommended that users interact with the program for approximately two hours per week, to ensure sufficient exposure to the content but also allow enough time to apply techniques “offline” in relevant circumstances.

#### CAU/WL group

Participants of the CAU/WL group will not receive the *Vorvida* intervention for the time of the study. However, they will receive a free access to *Vorvida* six months after registering for the study and will then also be able to use the program for another six months.

Participants of both groups are free to seek any other help they need or desire, e.g. pharmacological, psychological treatment or counselling.

### Assessments

We will conduct a total of three assessments. The first will be the baseline assessment (t0) before the randomization and the start of the intervention. The second and primary outcome will be three months after the randomization and the start of the intervention (t1) and the third will be after six months when the intervention is completed (t2) (see Table 2). All assessments will be conducted as self-reports via online-questionnaires, using a secure and widely used external online survey collection service (www.surveymonkey.com). The links to the questionnaires will be e-mailed to the participants.

**Table 2:** Measures and measurement points

	AUDIT-C	QFI^1^	TFB^2^	Being Drunk^3^	Binge Drinking^3^	CAEQ^4^	AASE-G^5^	RCQ-G^6^	Readiness-Ruler^7^	ZUF-8^8,9^
Screening	X	X								
t0 baseline		X	X	X	X	X	X	X	X	
t1 three month follow-up		X	X	X	X	X	X	X	X	X
t2 six month follow-up		X	X	X	X	X	X	X	X	X

^1^Quantity- Frequency-Index (amount of alcohol in g) last 30 days, ^2^Timeline-Follow-Back (amount of alcohol in g) last 7 days, ^3^Single item, ^4^ The Comprehensive Alcohol Expectancy Questionnaire, ^5^Alcohol Abstinence Self-efficacy Questionnaire, ^6^Readiness to change Questionnaire, Readiness-Ruler, ^8^Patient satisfaction questionnaire, ^9^Intervention group only

### Primary outcome measures

***Measurement of alcohol consumption*** will be the *Quantity*- *Frequency*-*Index* (*QFI*) [40–42] and the *Timeline*-*Follow*-*Back* (*TFB*) method (amount of alcohol in gram) last 7 days. For the QFI the frequency of consumption and quantity of consumption per drinking day within the last 30 days will be measured. Based on beverage-specific alcohol content (beer, wine/sparkling wine, spirits and mixed drinks) the content of pure alcohol in gram will calculated [43]. With the TFB method the respondents are asked to recall their alcohol consumption within the past seven days. Both methods are described with advantages and disadvantages, e.g. in the QFI method respondents are likely to underestimate their drinking whereas the TFB approach does not capture the drinking behavior of infrequent drinkers [41]. For this reason both approaches will be combined in this study.

### Secondary outcome measures

***Drinking behavior*** will be assessed by one item asking for drunkenness (on how many days within the past 30 days did you feel drunk (e.g. unsteady on the feet, blurred vision)?) and one item asking for binge drinking (on how many days did you drink more than five drinks one occasion?). These items were based on the questions developed for the project “Standardizing Measurement of Alcohol-Related Troubles” (SMART) [44].

***Expectancies of effects of alcohol use*** will be measured with the short form of the "Comprehensive Alcohol Expectancy Questionnaire" (CAEQ) [45], a self-assessment instrument with 19 items asking for the expectation of effects of alcohol in terms of (a) social Assertiveness and positive affect (b) tension reduction (c) cognitive impairment and physical discomfort (d) aggression, and (e) sexual enhancement. Items can be rated on a 5-point Likert-scale ranging from 1 (not at all) to 5 (definitely). The CAEQ has been found as a psychometrically sound tool.

***Abstinence and relapse*** will be assessed with the *Alcohol abstinence self-efficacy scale - German Version (AASE-G)* the German version of the alcohol abstinence self-efficacy questionnaire contains two scales similar to the original English version [46]. The scales confidence and temptation, each consisting of the same 20 items that can be rated on a 5-point Likert scale. The items capture the temptation to drink in a given situation and the confidence not to drink in this situation. The instrument measures self-efficacy expectations of being able to withstand drinking alcohol, as well as self-perceived risk. For the AASE solid subscale structure and strong indices of reliability and validity were demonstrated.

***Self-efficacy and motivation to change*** is captured with two measures.

First, the *Readiness to Change Questionnaire* – German version (RCQ-G) the German translation of "Readiness to Change Questionnaire" [47, 48] will be used. The RCQ-G consist of 12-items on a 5-point rating scale ranged from to “strongly disagree” (−2) to “strongly agree” (+2). The self-assessment on the RCQ-G allocates persons to three stages of readiness to change (pre-contemplation, contemplation, action). The RCQ-G shows satisfying psychometric properties in a German population of high-risk drinkers behavior [48].

Second, the adapted German version of the Readiness-Ruler form Demmel (2005) [47, 49] will be applied. Participants will be instructed to rate two items on an 11-point Likert scale. 1) on the *importance ruler* they will be ask to rate how important it is to them to change their alcohol drinking behavior, 2) on the *confidence ruler* they estimate how confident they are about changing their behavior. Both scales range from 0 (=not important at all/not confident at all) to 10 (=very important/very confident).

**Satisfaction** with the intervention is captured with the *Patient satisfaction questionnaire (ZUF-8)* which has been tested as an economical and reliable instrument [50, 51] that assessed satisfaction with inpatient treatment. It consists of 8 items with four options to answer. The measure was adapted to assess the satisfaction with an Internet intervention.

***Additionally,*** key demographic data (e.g. sex, age, employment status) will be assessed including information about the use of other treatment options (e.g. outpatient/inpatient counseling, psychiatrist, psychologist, self-help group)*.* Furthermore, we will inquire about the first time of alcohol consumption and the start of regular use of alcohol [40].

### Statistical analysis

Descriptive statistics will be calculated for demographic data and the primary and secondary outcomes by treatment allocation.

To test the effectiveness of the web-based self-help program *Vorvida* the primary outcome will be the reduction in alcohol consumption of the *Vorvida* group compared to the CAU/WL control group. Therefore, a baseline adjusted linear-mixed model will be calculated to measure the change in alcohol consumption from the baseline to the follow-up measure. The group will be defined as fixed factor using the baseline variable as control.

The intention-to-treat (ITT) analysis of primary data will be based on all available data from all randomized participants. For the primary outcome, an analysis of covariance (ANCOVA) will be calculated for the difference between the intervention and control group at t1 with group and baseline values as fixed effects. An additional analysis will be conducted on a per-protocol analysis set. However, sensitivity analyses will be performed with different methods of missing value imputation to study the robustness of the findings. Only the result of this primary effectiveness efficacy analysis will be interpreted in a confirmatory manner.

The secondary outcomes will be analysed using a baseline adjusted linear mixed model with group and particular baseline values as fixed effects and time as repeated effect. Therefore, on the lowest hierarchical level autocorrelation and heteroscedasticity will be assumed.

The two-sided α-level will be set at 0.05.

### Ethics

The study is being conducted in Compliance with the Declaration of Helsinki [52]. Approval was obtained from the Ethics Committee of the State Chamber of Physicians in Hamburg, Germany (reference number: PV4802).

## Discussion

The aim of this randomized-controlled trial will be to evaluate the effectiveness of a newly developed German Internet intervention, named *Vorvida,* which aims to help adults reduce their problematic alcohol consumption. *Vorvida* is, to our knowledge, the first dialogue-based Internet intervention for alcohol consumption reduction that uses an extensive tailoring approach to convey CBT content to users. Studies on a similar designed Internet self-help program for depression, named *Deprexis,* showed consistently good effectiveness, replicated in six RCTs to date [26, 53–56]. Moreover, *Vorvida* uses a fully responsive web-design approach, permitting users to switch between different devices, including smartphones, while continuing within the program wherever they left off on another device. We anticipate that the increased flexibility afforded by these technological features will translate into higher effectiveness compared to less flexible interventions that have been studied in other trials. This study is of particular interest because there is still a lack of RCTs on the effectiveness of extended and tailored Internet interventions for adults with harmful drinking or alcohol dependence [16].

There are also some limitations that ought to be considered. First, our study will only include problem drinkers with Internet access and moderate Internet literacy who are willing to participate and be randomized to a study condition. This will result in a sample bias in the sense that the sample cannot be expected to be representative of the general population of all problem drinkers, regardless of their Internet affinity or ability to use computer programs. However, the intended audiences for this intervention are clearly adults who are motivated and able to engage with Internet programs; therefore, the sample can be expected to be representative of the relevant population. In short, the results of this trial might suggest that *Vorvida* is effective for problem drinkers who can and wish to use Internet programs, although it will not inform the question of whether the program would also be effective for all persons with problematic drinking patterns, including those who cannot or do not want to use Internet-based programs.

It should also be noted as a limitation that Internet-based studies often suffer from high attrition, the “phenomenon of participants dropping out of eHealth trials” [57]. Efforts are made here to reduce attrition, though, as participants receive a gift voucher if they fill out all questionnaires from t1 to t2.

An additional limitation is that there will be no face-to-face diagnostic interviews administered by a qualified professional. Interested persons will be included or excluded based on their self-reported screening questionnaire filled in online. This limitation is explained primarily by limited resources, although it is also possible that initial face-to-face contacts can actually boost the effects achieved by Internet interventions, complicating the interpretability of such trials [27, 54].

Another limitation is that only self-report measures will be used, which introduces potential biases inherent in self-report questionnaires, such as social desirability. However, previous studies suggest that self-assessed methods in alcohol research tend to be reliable and valid [58, 59]. However, we acknowledge that the validity of the outcome measures used here has not explicitly been tested for online settings. Finally, a limitation is that we do not ask participants to provide open feedback about the program, but we do ask for the intervention groups’ satisfaction with *Vorvida*, using validated questionnaires.

In addition to these methodological limitations, it is important to note how potential suicidality among participants will be addressed. If suicidal tendencies are detected (e.g., because participants select relevant questionnaire responses), we will exclude the respective person from the study. Support numbers and contact addresses will be provided only through our study website. The only personal contact with a participant will occur if a participant will call the research investigators concerning any questions about the study procedure.

## Conclusions

If the results from this trial show effectiveness for the Vorvida program, this would suggest that this Internet intervention could be recommended for adults who seek help to overcome problematic drinking. This would not mean, though, that the program could serve as an alternative to established treatments, as such a comparison is not part or purpose of this trial. If shown to be effective, the Internet-based program *Vorvida* could be widely implemented to increase the accessibility of helpful techniques for problem drinkers who desire such help. It could be implemented in outpatient settings (e.g. general practitioners offices, treatment centers, welfare organizations) or inpatient settings. If the effects of this intervention will be found to be of a magnitude that is clinically relevant or relevant from a public health (population-based) perspective, this intervention could be used to reduce the existing treatment gap for alcohol-related disorders and thereby improve the quality of care for those affected by alcohol misuse.

### Trial status

Currently recruiting (N_current_ = 340 as of December 15^th^).
